# Tripartite motif containing 35 contributes to the proliferation, migration, and invasion of lung cancer cells *in vitro* and *in vivo*

**DOI:** 10.1042/BSR20200065

**Published:** 2020-04-23

**Authors:** Jingtao Zhang, Zihao Xu, Boyao Yu, Jiatang Xu, Bentong Yu

**Affiliations:** 1 Department of Thoracic Surgery, The First Affiliated Hospital of Nanchang University, Nanchang, Jiangxi, 330006, China; 2 School of Public Health, Nanchang University, Nanchang, Jiangxi 330006, China; 3 Xiangya School of Medicine, Central South University, Changsha, Hunan 410013, China; 4 The Second Clinical Medical College, Nanchang University, Jiangxi 330006, China

**Keywords:** invasion, migration, NSCLC, proliferation, TRIM35

## Abstract

The tripartite motif (TRIM) family is a family of proteins with highly conserved domains. Previous researches have suggested that the members of TRIM family proteins played a crucial role in cancer development and progression. Our study explored the relationship between TRIM35 and non-small cell lung cancer (NSCLC). The study showed that the expression of TRIM35 was increased in NSCLC samples, and patients with high expression of TRIM35 had a poor clinical prognosis. Overexpression of TRIM35 in NSCLC cell line H460 promoted cell proliferation, migration, and invasion, knockdown of TRIM35 produced an opposite result in A549 and H1299 cell lines. *In vivo* study further confirmed that overexpression of TRIM35 promoted tumor formation. The RNA-seq analysis suggested that TRIM35 might promote lung cancer proliferation, migration, and invasion by regulating cancer-associated functions and signaling pathways. Hence, we identified TRIM35 played a significant role in tumoral growth and was a potential diagnosis and prognosis target for lung cancer.

## Introduction

Lung cancer is an important cause of cancer-related deaths worldwide. In recent years, lung cancer has leaped to the highest morbidities and mortality rates among all cancers [1,2]. Among the different subtypes of lung cancer, non-small cell lung cancer (NSCLC) was the major subtype, accounting for approximately 80–85% of cases [3–5], with 2.1 million new lung cancer cases and 1.8 million deaths predicted in 2019 [6]. Although the treatment of lung cancer has made great breakthroughs in recent decades, traditional chemotherapeutic drugs lacked specific targeting specificity for NSCLC, or had toxic side effects on patients and limited efficacy [7]. The result of the patients’ long-term survival did not show significant improvements [8,9]. Therefore, it was necessary and urgent to explore new therapeutic targets for NSCLC.

Tripartite motif (TRIM) proteins were the member of the RING family of E3 ubiquitin ligase, including RING-finger domain, Zinc-binding domain (B-boxes domain), Coiled-coil domain, and C-terminal domain, and the presence of RING finger indicated that TRIM protein as E3 participated in various physiological processes in cells [10–13]. Previous studies had reported the important functions of TRIM proteins in the development of various cancers, which played an important role in cell cycle, apoptosis, differentiation, metabolism, and immune response [14–17]. The clinical manifestations observed in human diseases caused by TRIM gene mutations might be due to change in TRIM activity during ubiquitination [18,19]. Currently, TRIM35 has been found to act as a tumor-promoting gene in hepatocellular carcinoma and erythroleukemic, and it could regulate erythroid differentiation by modulating GATA-1 activity [20–22]. Yao et al. found that the down-regulation of TRIM35 expression promoted oncogenic activity in liver cancer cells [23]. Song et al. found that miR-4417 targeted TRIM35 to regulate the proliferation and apoptosis of liver cancer cells [24]. Peter et al. found increased expression of TRIM35 in HeLa cells inhibited cell growth, clonogenicity, and tumorigenicity [25]. However, the role of TRIM35 in NSCLC remained unclear, thus driving us to investigate the relationship between TRIM35 and NSCLC.

Our study found that TRIM35 was highly expressed in NSCLC tissues and related with lymph node. Besides, TRIM35 was found to act as an oncogene via effecting the migration, invasion, and proliferation of NSCLC cells.

## Materials and methods

### Cell culture

The NSCLC cell lines H1299, H460, H3122, HCC-827, A549 and human bronchial epithelial cells (HBE) were obtained from ATCC (Manassas, VA, U.S.A.). All of the cells were cultured in F12/DMEM (Gibco, U.S.A.) supplemented with 10% fetal bovine serum (Gibco, U.S.A.) and incubated at 37°C with 5% CO_2_.

### Immunohistochemistry

Paraffin-embedded specimens in approximately 10% formalin fixative buffer were cut into 4-μm-thick sections. Then we stained the sections with TRIM35 antibodies using standard immunohistochemical methods (dilution 1:200, Santa Cruz Biotechnology, U.S.A.). DAB systems detected positive staining cells and Hematoxylin was applied for counterstain.

### RNA extraction and RT-qPCR

Total RNA was extracted from cells using Total RNA Extraction Kit (Tiangen, China). PrimeScript RT reagent Kit (TaKaRa, Japan) was used to reverse transcribe the purified 0.5 g RNA. The resulting cDNA was used as a template for qPCR, and qPCR was performed by using SYBR Green to detect the level of mRNA. The primer sequences for PCR were listed in Table 1.

**Table 1:** The primer sequences for PCR

Gene	Forward primer (5′–3′)	Reverse primer (5′–3′)
*TRIM35*	CATCGCCAAGCACAATCAGG	GCGTTTTCGGCTCTTGTGTT
*E-Cad*	TTCTGCTGCTCTTGCTGTTT	TGGCTCAAGTCAAAGTCCTG
*N-Cad*	TGCGGTACAGTGTAACTGGG	GAAACCGGGCTATCTGCTCG
*NRP2*	CTGGAAGTCAGCACTAATGGAGAG	GCATCGTTGTTGGCTTGAAATACC
*FOXQ1*	ACG CTG GCG GAG ATC AAC GAG	AGG TTG TGG CGC ACG GAG TT
*NDRG1*	CTGCACCTGTTCATCAATGC	AGAGAAGTGACGCTGGAACC
*EGR1*	TTCGACCTGCTCATCTTCGG	CGATGCGTGAGTCCATGTGT
*PER1*	CTGCTACAGGCACGTTCAAG	CTCAGGGACCAAGGCTAGTG
*CLDN3*	GTCCGTCCGTCCGTCCG	GCCCAGCACGGCCAGC
*SOX2*	TCAGGAGTTGTCAAGGCAGAG	AGAGGCAAACTGGAATCAGGA
*CASC2*	GCACATTGGACGGTGTTTCC	CCC AGTCCTTCACAGGTCAC
*ID4*	GTGCGATATGAACGACTGCT	CAGGATCTCCACTTTGCTGA
*ANXA3*	CCCATCAGTGGATGCTGAAG	TCACTAGGGCCACCATGAGA
*GAPDH*	GTATGACAACAGCCTCAAGA	GTCCTTCCACGATACCAAAG

### Lentivirus packaging and cell transfection

pSin-puro-TRIM35 and pSin-puro-empty vector were purchased from Shanghai GeneChem Company. A total of three vectors (pSin-puro-TRIM35, pSin-puro-empty vector, and pMD.2G) were co-transfected into 293T cells using Lipofectamine 2000 (Millipore, United States). Virus supernatant collected after 72 h and stored at −80°C. H460 cells were cultured at a density of 30% in six-well plates, and 24 h later, the collected virus supernatant was added, and 8 μg/ml of Polybrene was added to increase the virus infection efficiency. The strain was obtained by screening with puromycin.

### Cell count kit-8

Transfected lung cancer cells were seeded into 96-well plates at a density of 1500 cells/well and three multiple holes in each group. After 24 h of cultivation, Cell Count Kit-8 (CCK-8) solution (Beyotime Biotechnology, China) was added and incubated at 37°C for 1 h and measured at an optical density (OD) of 450 nm. Continuously measured for 3 days and counted the results.

### Colony formation assays

Cells were seeded into six-well plates at a density of 250 cells/well, and each group had three duplicate wells. Cells were continuously cultured for 15 days in an incubator. After 15 days, the cloned cells were stained with 1% Crystal Violet for 30 min and counted under a microscope.

### Wound healing assay

Transfected tumor cells were added in a 12-well plate until the density is approximately 60%. The cell scratches were made by a P-200 pipette tip and photographed under a microscope. Cultured with 1% FBS and photographed under the microscope after 24 and 48 h, respectively.

### Transwell experiments

Tumor cells were seeded into 10-cm dishes 48 h before the experiment with 2 × 10^5^ cells per port. Serum-free medium was replaced 16 h before the experiment so that cells were starved. Matrigel was added to the upper transwell chamber (BD, Bedford, MA). A total of 3.5 × 10^4^ cells were seeded in a 24-well migration and added 0.8 ml of 10% serum-containing medium to the lower chamber. Cells were cultured in conventional cell culture conditions. After 24 h, fixed with 75% alcohol and stained with 1.2% Crystal Violet. Finally, three random fields were selected under the optical microscope (100×) and counted the cell numbers.

### Xenograft experiments

Four-week-old Balb/c female mice were obtained from the Nanjing University Laboratory Animal Company. All the experiments took place in the Nanchang University. H460 cells and overexpression-TRIM35 H460 cells were injected subcutaneously into mice at 1.5 × 10^7^/ml. All mice were killed after isoflurane anesthesia and tumor tissues were removed to observe and record tumor size and weight every other week. Procedures involving animals and their care were conducted in conformity with NIH guidelines (NIH Pub. No. 85-23, revised 1996). The Animal Experiment Ethics Committee of the First Affiliated Hospital of Nanchang University approved all animal experiments (approval number: SYXK2015-0001).

### Human clinical specimens

Ninety-two pairs of formalin-fixed, paraffin-embedded lung cancer, and adjacent tissue samples from patients undergoing lung cancer resection were analyzed at the First Affiliated Hospital of Nanchang University. Retrieved and analyzed patient clinical data, including age, sex, tumor size, TNM stage, and pathologic grade. Experiments using human materials were performed in strict accordance with Declaration of Helsinki. All materials were approved by the Ethics Committee of the First Affiliated Hospital Nanchang University. Informed consent was signed for all patients.

### RNA-Seq sample preparation and data analysis

H460 cells stably transfected with normal H460 cells and overexpression-TRIM35 H460 cells were cultured to a certain amount like the above normal cells. Reverse transcription into cDNA after extraction of total RNA and sequenced at the Shanghai Bohao Biotechnology Institute (Shanghai, China) using HiSeq 2500 platform.

### Enrichment analysis

The Database for Annotation Visualization and Integrated Discovery (DAVID) is an online annotation tool that provides a comprehensive understanding of genetic and protein biological information [26]. Kyoto Encyclopedia of Genes and Genomes (KEGG) pathway enrichment and Gene Ontology (GO) enrichment analysis evaluated the many functions and valuable pathways of differential gene (DEG). We used *P*<0.05 and (fold change) |FC| > 2 as a DEG requirement.

### Statistical analysis

SPSS 24.0 was used to perform all statistical analyses, numerical data expressed as mean ± standard error, and calculated using a two-tailed Student’s *t* test, chi-square test, and Spearman correlation analysis. *P*<0.05 was statistically significant.

## Results

### TRIM35 was highly expressed in lung cancer tissues and cell lines

**Figure 1:**
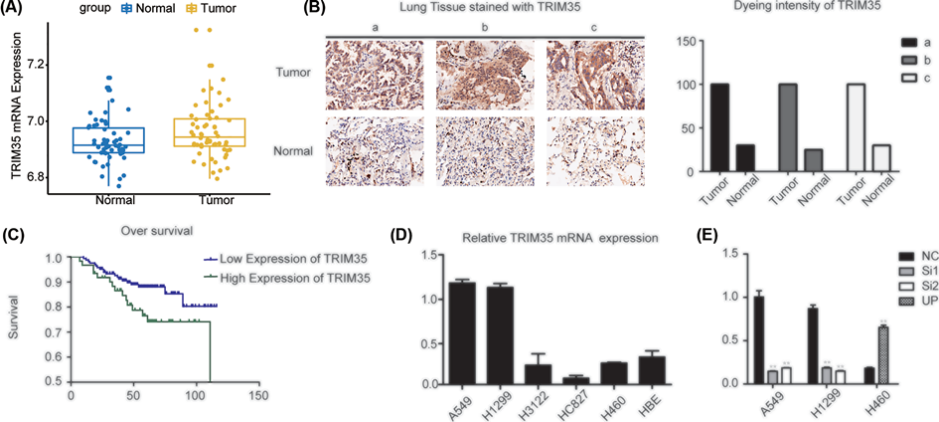
Expression of TRIM35 in lung cancer tissues and cell lines (**A**) TRIM35 expression was significantly increased in NSCLC tissues compared with non–tumor tissues in Oncomine database. (**B**) IHC staining of TRIM35 in NSLCL tissues and normal tissues and dyeing intensity of TRIM35 expression showed TRIM35 was significantly increased in NSCLC tissues. (**C**) Overall survival (OS) in higher or normal TRIM35 expression in NSCLC tissues. TRIM35 expression was negatively associated with overall survival in NSCLC. (**D**) TRIM35 expression was detected by qRT‐PCR assay in NSCLC cell line A549, H1299, H3122, HC827, and H460 cell lines and normal lung cell lines HBE. (**E**) TRIM35 mRNA levels after knocked down TRIM35 in A549 and H1299 cells and overexpressed TRIM35 in H460 cells. **, *P*<0.01. All data are presented as mean ± SD from three independent experiments. Lung cancer tissue samples (a,b,c).

We analyzed the expression of TRIM35 in lung cancer and normal tissues in the Oncomine database. The Oncomine showed that TRIM35 was highly expressed in lung adenocarcinoma (LUAD) (Figure 1A). To validate this result, immunohistochemistry (IHC) was used to determine the expression of TRIM35 in NSCLC tumor samples compared with adjacent normal tissues. These results indicated that TRIM35 was highly expressed in NSCLC tissues (Figure 1B). Besides, we analyzed the expression of TRIM35 in lung cancer tissues from 92 patients clinically surgically resected tumor and revealed the relationship between TRIM35 gene expression level and lung cancer pathological type, TNM stage, tumor size, gender, age etc. (Table 2). The results indicated that high expression of TRIM35 was associated with metastasis of NSCLC especially in lymph node (*P*=0.011). Furthermore, we analyzed the relationship between TRIM35 expression and prognosis in lung cancer. Results showed that patients with high TRIM35 expression had poor clinical prognosis (Figure 1C). Finally, we analyzed the expression of TRIM35 in five lung cancer cell lines (A549, H1299, H3122, H460, HCC-827) and normal lung epithelial cells (HBE). It was found that TRIM35 was significantly higher in lung cancer cells than HBE cells (Figure 1D). According to the expression of TRIM35, we transfected siRNAs into A549 and H1299 or overexpression for H460 (Figure 1E).

**Table 2:** Correlation between TRIM35 expression and clinicopathological characteristics

	Variables	TRIM35 expression		Total	χ2	*P*-value
		High	Low			
Age (years)					1.911	0.167
	≤60	6	36	42		
	>60	13	37	50		
T stage					0.868	0.351
	T1/T2	16	54	70		
	T3/T4	3	19	22		
Sex					1.447	0.229
	Female	11	31	42		
	Male	8	42	50		
TNM stage					1.733	0.188
	I/II	12	34	46		
	III/IV	6	35	41		
	Null			5		
N stage					6.443	0.011
	N0	11	28	39		
	N1/N2/N3	2	33	35		
	Null			18		
M					0.267	0.605
	M0	19	71	90		
	M1	0	1	1		
	Null			1		
EGFR					1.672	0.196
	Negative	17	55	72		
	Positive	1	12	13		
	Null			7		
Grade					1.518	0.218
	I	3	5	8		
	II/III	16	68	84		

### TRIM35 promoted the proliferation of lung cancer *in vitro* and *in vivo* experiments

**Figure 2:**
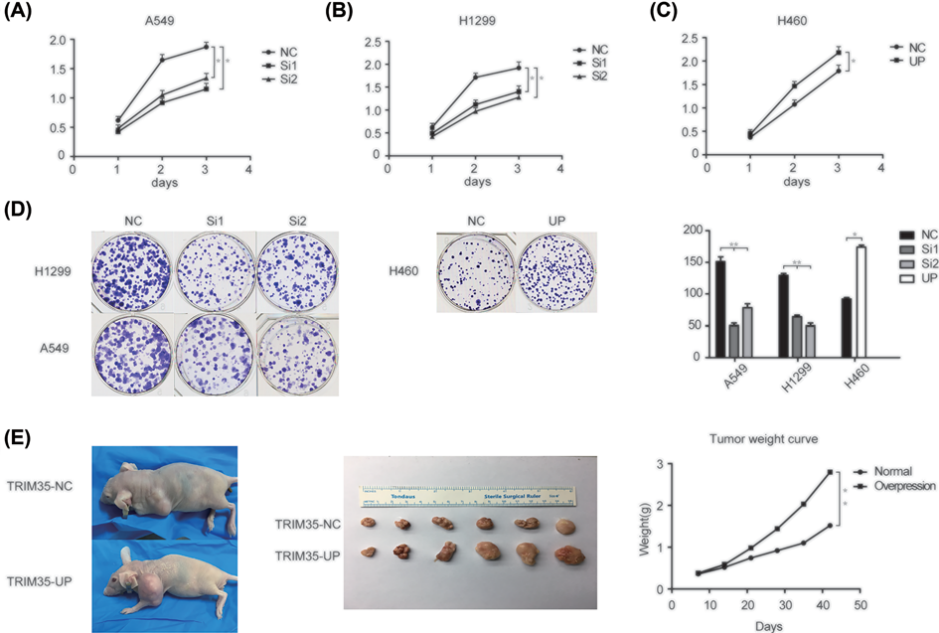
TRIM35 promotes lung cancer cell proliferation *in vitro* and *in vivo* As detected by CCK-8 assay, the silencing of TRIM35 reduced (**A**) A549 and (**B**) H1299 cell proliferation. (**C**) Up-regulation of TRIM35 enhanced H460 cell proliferation. (**D**) Colony formation assays showed that colony formation was dramatically reduced following TRIM35 was silenced, and increased when TRIM35 was up-regulated. (**E**) Enhanced tumorigenicity of NSLCL cells *in vivo* by TRIM35 overexpression. The tumor weight of the overexpression group was higher than the normal group. *, *P*<0.05, **, *P*<0.01. All data are presented as mean ± SD from three independent experiments.

CCK-8 assays used to investigate the effects of knockdown and overexpression of TRIM35 on cell proliferation. It was found that the proliferative ability was significantly weakened after knocking down TRIM35 in A549 and H1299 cells (Figure 2A,B), and the proliferation ability was enhanced after overexpression of TRIM35 in H460 (Figure 2C). Colony formation assays were also showed the role of TRIM35 in promoting the proliferation of A549, H1299, and H460 cells (Figure 2D). To further validate the role of TRIM35 *in vivo*, we constructed a stable H460 cell overexpressing TRIM35 with lentivirus and cultured to a sufficient number. We divided 12 female mice with 4-week-old severe immunodeficiency into two groups. Normal H460 cells and H460 cells overexpressing TRIM35 were inoculated into the two groups of mice. One mouse from each group was killed every other week and weighed. It was found that the size of tumors of overexpressing TRIM35 group was significantly heavier than the normal group (Figure 2E).

### TRIM35 promoted migration and invasion of lung cancer cells

**Figure 3:**
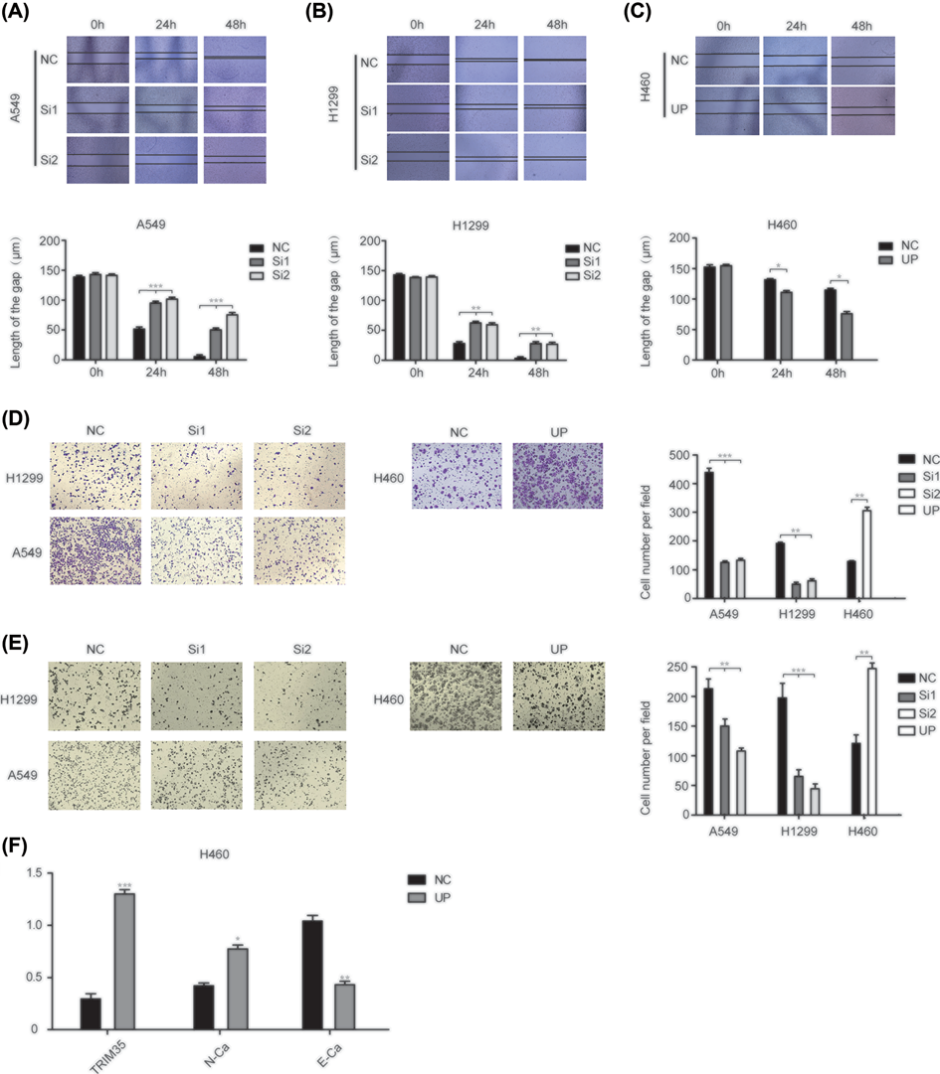
Effects of TRIM35 on NSCLC cell migration and invasion Scratch test verified that the migration of (**A**) A549 and (**B**) H1299 cells was inhibited after silencing TRIM35. (**C**) Scratch test showed up-regulating TRIM35 promoted migration of H460 cells. (**D**) Transwell assays without Matrigel indicated that overexpression of the TRIM35 can promote the migration of H460 cells while inhibiting the expression of the TRIM35 can suppress the migration of A549 and H1299 cells. (**E**) Transwell assays with Matrigel indicated that overexpression of the TRIM35 enhanced the invasion ability in H460 cells and inhibiting the expression of the TRIM35 presented the reverse effects in the A549 and H460 cells. (**F**) The result of E-cad and N-cad expression of mRNA indicated overexpression of TMRM35 could promote epithelial–mesenchymal transition (EMT) in NSCLC cell lines. *, *P*<0.05, **, *P*<0.01, ***, *P*<0.001. All data are presented as mean ± SD from three independent experiments.

To further determine the effects of TRIM35 on lung cancer cell migration and invasion, we first performed Wound healing assays on A549, H1299, and H460 cells. At 0, 24, and 48 h, it was found that the migration ability was weakened after knocking down TRIM35 in A549 and H1299, and the migration ability was enhanced after overexpressing TRIM35 in H460 (Figure 3A–C). Consistently, the transwell assays without Matrigel results showed that knocked down TRIM35 inhibited the migration capacity of A549 and H1299 cells, and up-regulation of TRIM35 increased the migration capacity of H460 (Figure 3D). And we found that A549, H1299, H460 cells showed a similar pattern when we added the Matrigel in the upper transwell chamber (Figure 3E). In view of the obvious effect of TRIM35 on the migration and invasion of lung cancer cells, we performed qPCR verification on E-Cad and N-Cad. It was found that N-Cad increased at the same time as TRIM35 increased, while E-Cad decreased. Above all, the TRIM35 can significantly regulate the migration and invasion ability in A549, H1299, and H460 cells *in vitro* (Figure 3F).

### GO term and KEGG pathway enrichment analysis

**Figure 4:**
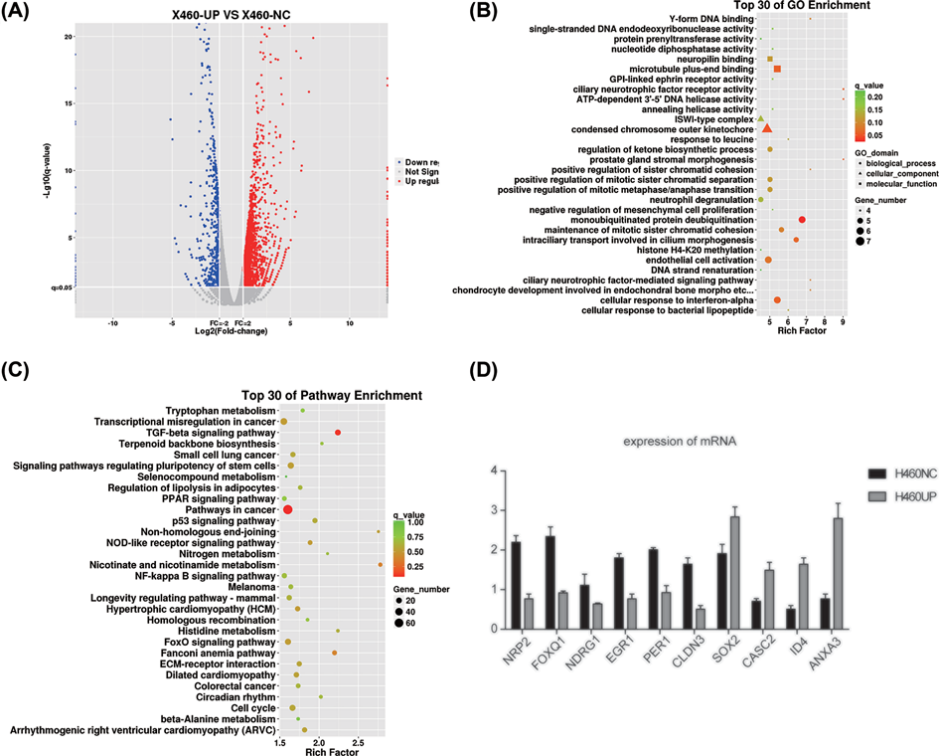
The result of RNA-seq (**A**) The volcano map showed that 2286 up-regulated genes and 385 down-regulated genes in X460-UP cells in comparison with X460-NC. It was considered to be statistically significant when Log2 fold change (Log2FC) > 1 or < −1, and *P*<0.05. The top 30 enriched (**B**) GO terms and (**C**) KEGG pathways. Gene number: number of target genes in each term or pathway. Rich factor: the ratio of the number of target genes divided by the number of all the genes in each term or pathway. (**D**) Several genes were selected to verify whether the expression changes were consistent with the volcanic map.

In order to find molecules or pathways that may work together with TRIM35, we used the h460 cell line expressing TRIM35 and normal h460 cells for RNA-seq analysis, and found 2858 differentially expressed genes, of which 2286 genes were highly expressed and 572 genes were underexpressed (Figure 4A). The differentially expressed genes are mapped to each item in the GO database, and the number of genes in each item is calculated. Then, using the hypergeometric test, compared with the whole genome background, the GO entries with significant enrichment in the differentially expressed genes are screened. The KEGG pathway enrichment analysis of DEGs was performed using the same principle (Figure 4B,C). Finally, we selected the more studied molecules for qPCR and found that NRP2, FOXQ1, NDRG1, EGR1 PER1, CLDN2 were down-regulated with TRIM35 expression up-regulation, while SOX2, CASC2, ID4, ANXA3 were up-regulated with TRIM35 expression up-regulation (Figure 4D). So, we suspect that TRIM35 may work with one or more of these genes.

## Discussion

As the world’s highest malignant tumor, lung cancer has made great breakthroughs in its diagnosis and treatment in recent years, but the current survival rate of lung cancer patients is far from satisfactory [27–29]. In order to improve the clinical prognosis of lung cancer, it is particularly important to find effective anti-tumor strategies. The development of lung cancer is a multistep process characterized by a series of genetic changes. Although many studies on lung cancer genes have been done before, the function of most genes remains unclear. The TRIM protein family plays a vital role in cellular processes and is closely related to the development and prognosis of many cancers [20,21]. TRIM35 is located at chromosome 8p21 and encodes a protein of 493 amino acids [25]. According to previous studies, TRIM35 has both cancer-promoting and tumor-suppressing effects. For example, HBV-miR-2 inhibits TRIM35 expression by targeting its 3′-untranslated region (3′UTR) [23]. TRIM35 suppresses the tumorigenicity of liver cancer cells through the blockade of PKM2/Y105 phosphorylation [30]. And the expression of TRIM35 suppressed the tumorigenic phenotype of HeLa cells [25]. These results indicated the importance of TRIM35 in tumorigenesis and development. But the role of TRIM35 in the NSCLC process is still unknown. In order to study the mechanism of TRIM35, we found that the expression of TRIM35 in lung cancer and normal tissues were not significantly different by database analysis. This result is contrary to what we expected, and then by IHC, TRIM35 is found in the lung cancer cytoplasm. The expression was significantly up-regulated, and then we performed a series of phenotypic experiments. The overexpressing-TRIM35 cell lines were constructed in H460 which TRIM35 overexpress at a lower expression, and the expression of TRIM35 was knocked down in the higher expressing A549 and H1299 cell lines. Based on the results of CCK-8 and clone formation, we have seen the significant role played by TRIM35 in lung cancer proliferation. And we found overexpressed TRIM35 promoted tumor formation in nude mice *in vivo*. After this we further validated the effect of TRIM35 on lung cancer cell migration and invasion by scratch and transwell experiments. These results demonstrated that TRIM35 promotes tumorigenesis and progression. To explore the possible functions of TRIM35, by performing GO and KEGG enrichment analysis of DEGs. The differentially expressed genes are mapped to each item in the GO database, and the number of genes in each item is calculated. Then, using the hypergeometric test, compared with the whole genome background, the GO entries with significant enrichment in the differentially expressed genes are screened. The KEGG enrichment of DEGs was performed using the same principle. For cell component (CC), the DEGs were enriched in condensed chromosome outer kinetochore and ISWI-type complex, the DEGs were particularly enriched in biological processes (BPs), including monoubiquitinated protein deubiquitylation, intraciliary transport involved in cilium morphogenesis, and cellular response to interferon-α. For molecular function (MF), the DEGs were enriched in ciliary neurotrophic factor receptor activity and prostate gland stromal morphogenesis. The most significantly enriched pathways of DEGs were analyzed by KEGG analysis, including the TGF-β signaling pathway and pathways in cancer.

Epithelial–mesenchymal transition (EMT) is a complex molecular program that contains proteins such as E-cadherin and N-cadherin. There are related to fibroblast-like morphological changes in tumor cells [31–33]. Related studies have found that they are involved in the progression of lung cancer as an important role [34–36]. During the research, we found TRIM35 promoted the migration and invasion of lung cancer cells. We tested the mRNA expression of E-cadherin and N-cadherin, and the results were as expected. We suspect that TRIM35 promotes the migration and invasion of lung cancer cells through the EMT process.

In summary, our study established TRIM35 as a new tumor-promoting factor in lung cancer and its loss is sufficient to inhibit lung cancer *in vivo* and *in vitro*. Finally, our study highlights the potential of TRIM35 as a diagnostic and prognostic marker for lung cancer.

## Abbreviations

CCK-8: Cell Count Kit-8
DEG: differential gene
EMT: epithelial–mesenchymal transition
GO: Gene Ontology
HBE: human bronchial epithelial cell
IHC: immunohistochemistry
NSCLC: non-small cell lung cancer
TRIM: tripartite motif
